# A Meta-Analysis on the Impact of the Supplementation of Rumen-Protected Choline on the Metabolic Health and Performance of Dairy Cattle

**DOI:** 10.3390/ani9080566

**Published:** 2019-08-16

**Authors:** Elke Humer, Geert Bruggeman, Qendrim Zebeli

**Affiliations:** 1 Institute of Animal Nutrition and Functional Plant Compounds, Department for Farm Animals and Veterinary Public Health, University of Veterinary Medicine Vienna, 1210 Vienna, Austria; 2 Nutrition Sciences, Booiebos 5, 9031 Drongen (Gent), Belgium

**Keywords:** cow fertility, cow health, meta-analysis, milk production, rumen-protected choline

## Abstract

**Simple Summary:**

During the first weeks of lactation, dairy cows typically experience negative energy balance, leading to the mobilization of energy reserves. This predisposes early lactating cows towards metabolic diseases, such as fatty liver syndrome and ketosis. The supplementation of rumen-protected choline (RPC) is a strategy to restrict negative effects associated with negative energy balance in early lactating cows, but reported effects are inconsistent. This meta-analysis revealed that the supplementation of RPC positively affected dry matter intake, but this effect was associated with increased milk yield, thus without improving energy balance and metabolic profile of the cows.

**Abstract:**

After parturition, cows undergo negative energy balance leading to fat mobilization, predisposing them to fatty liver syndrome and ketosis with major consequences for health and reproduction. Supplementation of rumen-protected choline (RPC) has attracted major research efforts during the last decade, assuming that choline improves liver function by increasing very low-density lipoprotein exportation from the liver, thereby improving metabolic profiles, milk production, and reproduction. However, the effects of RPC on production, health, and reproduction have been inconsistent. Therefore, the aim of this meta-analysis was to evaluate the effects of RPC supplementation, starting from d 20 (± 12.2) ante partum to d 53 (± 31.0) postpartum, on feed intake, milk production performance and metabolic profiles of dairy cows early postpartum. Data analyses from 27 published studies showed an increase in postpartal dry matter intake (from on average 19.1 to 19.9 kg/d; *p* < 0.01) and milk yield (from on average 31.8 to 32.9 kg/d; *p* = 0.03) in cows receiving RPC. Milk fat yield and milk protein yield were also increased (*p* ≤ 0.05), without changing milk protein and fat contents. However, no interactive effects between cow’s milk yield level and RPC-supplementation as well as no dose-dependent effects of RPC supplementation were observed. Supplementing the diet with RPC showed no effects on blood metabolites (non-esterified fatty acids, beta-hydroxybutyrate, glucose, and cholesterol), independent of the milk yield level of the cows. An effect on liver triacylglycerol contents, incidence of ketosis, and mastitis could not be confirmed across all studies included in this meta-analysis. Also, the positive effects of RPC supplementation on reproductive performance were not consistent findings. In conclusion, supplementing RPC in lactating dairy cows showed positive effects on dry matter intake which likely caused the improved milk yield. However, RPC supplementation did not improve the metabolic health status of the cows. As several factors might be related to the responses to RPC, further research is needed to explore the precise mechanisms of RPC action in lactating cows, especially with regards to feed intake improvement and its related metabolic health-promoting potential in early lactating dairy cows.

## 1. Introduction

The transition period is critical for productivity, health, and fertility of dairy cows. Due to the reduced dry matter intake (DMI) around parturition, the energy intake of high-producing dairy cows is usually insufficient to meet the high energy needs for milk production, resulting in a negative energy balance (NEB; [1]). This leads to the mobilization of fat from adipose tissue, mainly in the form of non-esterified fatty acids (NEFA), which are released into the blood as a source of energy [2]. The limited capacity of the bovine liver to oxidize NEFA or to secrete esterified fatty acids (triacylglycerol; TAG) as very low-density lipoproteins (VLDL) predisposes early lactating cows toward hepatic dysfunction [1]. More specifically, the excessive amounts of NEFA overwhelm hepatic oxidation, leading to a ketotic state that is associated with increased production and secretion of ketone bodies, mainly beta-hydroxybutyrate (BHBA). Moreover, TAG accumulation in the liver enhances the risk of the cows experiencing “fatty liver” syndrome [2]. The elevated levels of NEFA and BHBA further contribute to oxidative stress, inflammatory responses, and a compromised immune system, therefore enhancing the susceptibility to infectious diseases and impairing fertility [3].

Choline represents a key nutrient for maintaining a normal concentration of fat in the liver due to its involvement in fatty acid transport [4]. More specifically, choline is an integral part of the structural component of tissues (e.g., phosphatidylcholine in cell wall membranes), mostly in the form of phosphatidylcholine and lysophosphatidylcholine, which play a vital role in cellular structure and activity [5]. Choline is also involved in the metabolism of fatty acids in the liver [3] and promotes the export of fat from the liver as part of VLDL. Therefore, choline is a major lipotropic compound in dairy cows, with the potential to decrease the concentration of hepatic TAG around parturition [6]. Because dietary choline is rapidly degraded by rumen microbes, which lead to only <20% bioavailability of choline [7], the only effective method of increasing choline availability to dairy cows is to feed it in a form that is protected from ruminal degradation [1]. In this regard, rumen-protected choline (RPC), containing choline chloride covered by a protective layer of fatty acids, has received interest as a feed additive [8].

The potential positive effects of choline on fat metabolism and milk production have attracted major research efforts on RPC supplementation in dairy cows during the last decades. The results about the effects of RPC on performance, metabolic health, and reproduction have been rather inconsistent. Sales et al. [9] conducted the only meta-analysis on the effect of dietary RPC on dry matter intake, milk yield and milk fat, and protein contents as well as yield including 11 studies that were available at that time. As several new studies were conducted from 2010 onwards, the aim of the present meta-analysis was to evaluate the current data with regards to the effects of RPC supplementation on a larger set of variables, including DMI, milk production responses, and also blood metabolites related to metabolic health in cows with various milk production level. A further aim was to describe the supplemental effects of RPC on postpartal diseases and reproductive performance in dairy cows.

## 2. Materials and Methods

### 2.1. Literature Search

A literature search was conducted using the public search generators Pubmed, Google Scholar, Web of Science, ScienceDirect, and Scopus. The main aim of the present study was to explore the impact of dietary RPC supplementation on productive performance, health, and reproduction. For that reason, research articles in scientific journals on controlled experiments investigating the effect of supplementation of RPC on the respective variables between the years 2000 and October 2018 and published in English were primarily considered for data extraction.

Stringent criteria were in place whether published experiments were included or excluded in this study. Quality assessment criteria included information about dietary composition, choline level and source (only studies using RPC were included), type of cows, lactation stage, parity, number of cows within treatment groups, duration of the experimental period, experimental design, description of statistical analysis, and parameters of interest (i.e., DMI, milk yield, milk constituents, blood metabolites, disease incidence, reproductive measures). A total of 27 studies were identified that fulfilled the required criteria. A summary of the studies used and the treatments, as well as the investigated parameters, are shown in Table 1.

Commercial products were used as dietary RPC supplements. A description of the products including product name, manufacturer, the content of choline chloride and rumen stability, is presented in Table 2.

Relevant descriptive statistics of DMI, milk production responses, and blood metabolites are summarized in Table 3.

### 2.2. Statistical Analyses

Descriptive statistics of the dependent variables were computed using PROC MEANS of SAS (version 9.4., SAS Institute, Cary, NC, USA). Statistical analysis of the performance and blood data of either control cows (CON) or cows supplemented with RPC was performed using PROC MIXED. The model included the random effect of the experiment, representing the variance between experiments accounted for by the other variables such as the physiological status of the animals, experimental design, and measurement methods. The variable “experiment” was classified in the CLASS statement. The number of animals was used to weight the means.

To evaluate a possible interactive effect between the RPC supplementation and the performance level of the cows, studies were grouped based on their cows’ milk level into low-producing cows (milk yield < 25 kg/d; *n* = 13 treatment means), medium-producing cows (milk yield >25 kg/d and <35; *n* = 27 treatment means), and high-producing cows (milk yield >35 kg/d; *n* = 31 treatment means) according to the reported treatment mean. The inclusion of RPC (CON vs. RPC), production level (low, medium, high) as well as their interaction were considered as fixed effects in the model.

Using a meta-regression approach, the effects of the choline chloride level as a continuous predictor on production performance parameters and blood metabolites were analyzed by PROC REG. Accompanying the regression predictors, RMSE was included.

A possible effect of the energy density of the diet, CP content, and content of fiber (i.e., NDF) was evaluated by including the respective variables as covariables in the statistical model. However, as none of these variables showed any interactive effect with the supplementation of RPC, they were not included in the final analyses.

## 3. Results

### 3.1. Effect of Rumen-Protected Choline on Dry Matter Intake and Milk Production Responses

The analysis of available data showed that the RPC supplementation did not affect DMI pre-partum, but supplementing RPC increased postpartal DMI by an average of 0.79 kg/d (*p* < 0.01; Table 4). The enhancing effect of RPC on DMI went along with an increased milk yield by on average 1.05 kg/d (*p* = 0.02). Also, the milk protein yield and milk fat yield were increased (*p* ≤ 0.05), while milk protein and fat percentage were not altered (*p* ≥ 0.78).

In general, no interactive effect between milk production level and RPC supplementation were observed (data not shown). Moreover, the DMI and milk production parameters were not influenced by the dietary RPC level. Parameters estimates obtained with the regression model are shown in Table 5.

**Figure 1:**
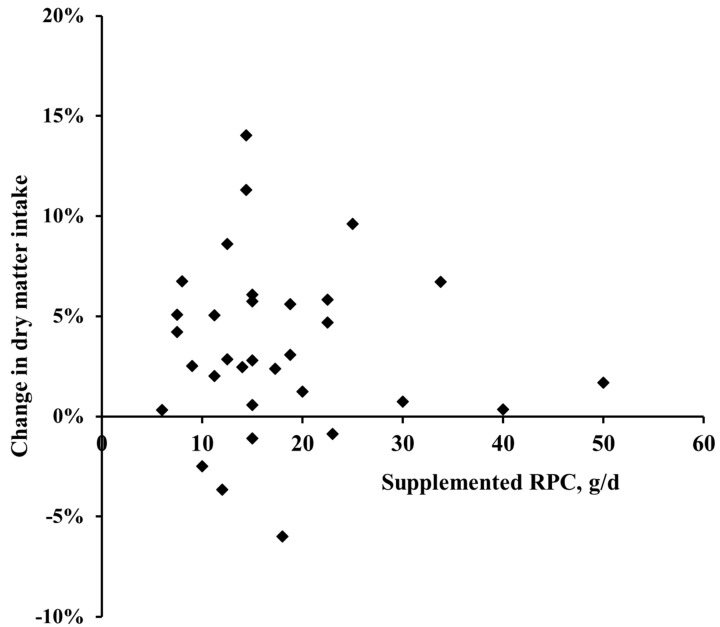
The relationship between the level of dietary supplementation with choline chloride in the form of rumen-protected choline (RPC) ranging from 6 to 50 g per cow per day and the relative change in dry matter intake compared to unsupplemented control cows reported by the studies summarized in Table 1.

**Figure 2:**
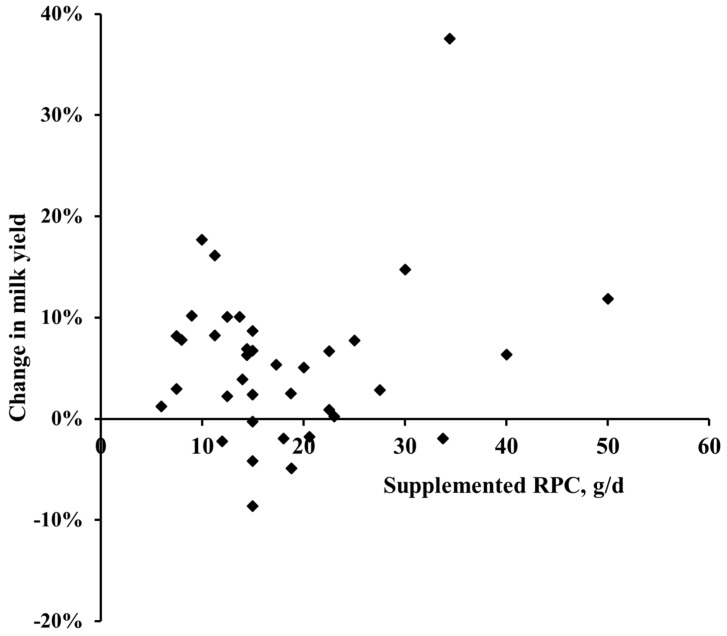
The relationship between the level of dietary supplementation with choline chloride in the form of rumen-protected choline (RPC) ranging from 6 to 50 g per cow per day and the relative change in milk yield compared to unsupplemented control cows reported by the studies summarized in Table 1.

Figure 1 and Figure 2 illustrate the relationship between the supplemental choline chloride levels and the relative change in DMI and milk yield, respectively. Overall, only in 5 cases, a numerical decrease in DMI (ranging from −0.9 to −6%) was observed, while the remaining 27 treatment means were increased by the RPC up to 14%. However, no clear effect of the dosage of choline chloride ranging from 6 to 50 g of RPC per day on the response in DMI was found. Similarly, the milk yield was numerically decreased in 8 treatments but increased in 29 cases up to 38% (Figure 2). Again, no dose-dependent effect of the choline chloride supplementation level was observed.

### 3.2. Effect of Rumen-Protected Choline on Body Weight and Body Condition Score

Several studies reported the effect of RPC on BW and body condition score (BCS). However, due to limitations in data quality, such as missing information on the BW after parturition or only graphical illustration of BW and BCS, statistical analysis of the respective variables was not feasible. An observation of the individual studies indicated that most of the studies found either no effect on postpartal BW and/or BCS [6,11,14,18,19,24,25,27,28,29,31,33,35], or even negative effects [10] of RPC supplementation.

### 3.3. Effect of Rumen-Protected Choline on Blood Metabolites

Table 4 summarizes the effects of RPC-supplementation on blood metabolites. Overall, the response of blood metabolites in cows to RPC supplementation has been inconsistent. Our meta-analytical approach revealed no effect of RPC supplementation on blood NEFA (*p* = 0.69). Also, for BHBA no overall lowering effect was observed in dairy cows supplemented with RPC (*p* = 0.74). In accordance, no effect of RPC on blood glucose and cholesterol was found (*p* ≥ 0.46).

Moreover, no interactive effect between milk production level and RPC supplementation on the blood metabolic profile was observed (data not shown). The regression between RPC doses and blood parameters are presented in Table 5. In general, the supplementation levels of RPC did not affect all investigated parameters.

### 3.4. Effect of Rumen-Protected Choline on Postpartal Disorders 

Table 6 summarizes the effects of RPC on postpartum disorders. In general, inconsistent effects on the risk of ketosis were observed. For instance, Lima et al. [36] observed improved health of early lactating cows in terms of reduced incidences of ketosis and mastitis when they were fed 15 g choline chloride in the form of RPC per day from 25 days before calving until 80 days in milk (DIM). When heifers received 15 g choline chloride per day only 21 days before calving until parturition, controversial effects on health were observed. More specifically, while RPC had no effect on the incidence of ketosis and mastitis, higher incidences of metritis and fever were observed. However, this group of cows showed an overall lower incidence of ketosis and mastitis compared to the former group, which received the RPC for a longer period of time. Also, Davidson et al. [18] found a lower ketosis incidence in multiparous dairy cows supplemented with 40 g of choline chloride in the form of RPC from 21 to 91 DIM, although no statistical analysis was possible due to the low number of observations. However, other studies observed no beneficial effect of RPC on the incidence of ketosis [6,11,14], whereby it has to be noted, that the overall ketosis risk in several studies was already low (e.g., [11,22,34]).

### 3.5. Effect of Rumen-Protected Choline on Reproduction

Inconsistent effects of RPC on reproduction have been reported (Table 7). While Ardalan et al. [22] and Furken and Hoedemarker [37] observed improved reproduction in cows receiving RPC, as demonstrated by increased cyclicity or pregnancy rates, Lima et al. [36] and Pirestani and Aghakhani [34] reported controversial effects of RPC on reproduction. More specifically, while Pirestani and Aghakhani [34] observed negative effects on RPC on reproduction, Lima et al. [36] found a higher proportion of multiparous cows to be cycling at 61 DIM, but the opposite effect in primiparous cows.

## 4. Discussion

The aim of this meta-analysis was to evaluate the current published data regarding the supplementation with RPC on DMI, milk production responses, metabolic health, postpartal diseases, and reproductive performance in dairy cows. While supplementing RPC showed no effect on prepartal DMI, an important finding of the present analysis was that it caused an increase in postpartal DMI by on average 0.79 kg/d. Taking the average energy and CP content of the lactation diets summarized in this meta-analysis (6.86 MJ NEL and 156 g CP per kg DM), this increase means a daily increase of about 5.4 MJ NEL and 123 g CP ingested, respectively. It is expected that this improved energy and nutrient intake has contributed to alleviate the NEB in early lactating cows. A low DMI after parturition is a major factor contributing to the severity of NEB and the enhanced risk of developing metabolic disturbances [1]. This alleviating effect is expected to be either observed as an improved NEB, i.e., less fat mobilization, reflected in less BW loss, lower circulating NEFA and BHBA, and greater glucose and cholesterol, or as increased milk yields. From the metabolic regulation perspective, however, the homeorhetic regulation during early lactation cows direct the metabolic fuels towards milk production instead of lowering the energy mobilization [42]. Indeed, supplementing RPC demonstrated to cause an increase in milk yield by on average 1.05 kg/d. Mechanistically, an improvement of energy intake with 5.4 MJ NEL would lead to the production of about 1.6 kg milk with 4% fat. Therefore, it is reasonable to assume that the enhanced DMI was responsible for the improved milk yield in RPC-supplemented cows.

Besides this indirect effect of RPC on milk yield, it has also been speculated that RPC can directly affect milk yield. A direct effect might derive from the lipotropic effect of choline in terms of promoting the export of fat from the liver, which has been previously suggested as the main mechanism responsible for the improvement of milk yield in cows supplemented with RPC around parturition [6]. A further direct effect might be the provision of methyl-donors, therefore providing higher levels of available methionine [3]. This is supported by findings of Davidson et al. [18], who observed that multiparous cows fed diets inadequate in methionine improved milk production when supplemented with RPC. Also, an indirect effect due to an improved general health status might be responsible for the enhancing effect on milk yield [18,36]. Furthermore, lower hepatic TAG might improve gluconeogenesis in the liver [43]. In this regard, Goselink et al. [43] observed elevated levels of glucose transporter 2 mRNA and a reduced peak in pyruvate carboxylase mRNA immediately after parturition in dairy cows supplemented with 14.4 g choline chloride in the form of RPC per day from 21 days ante partum to 42 DIM, suggesting improved carbohydrate metabolism in the respective cows. Due to these aspects, it could be speculated that higher yielding cows and cows with a higher risk for lipid-related disorders, might benefit more from RPC [13,18]. However, Pires and Grummer [44] suggested that the response to RPC does not depend on the production potential of the cows. This is also supported by the present finding, as no interactive effect between RPC supplementation and production potential was observed.

The increased milk yield caused an enhanced milk protein and fat yield. However, the increased yields of milk components from feeding RPC have been the results of increased milk yield, as the contents of milk fat and protein contents were not affected. Previously it has been suggested that choline might enhance milk fat synthesis as choline is used for phospholipid synthesis, thus facilitating lipid absorption and transport [45]. In general, in early lactating cows, the majority of milk fat derives from plasma NEFA. As RPC might reduce fat mobilization and thus reduce NEFA [12,16], potential effects of RPC on intestinal lipid absorption and hepatic TAG secretion and subsequent transport to the mammary gland might be obscured by the lowered availability of NEFA for milk synthesis [45]. It was also suggested that supplementary RPC would increase milk protein content due to the provision of methyl groups and methionine sparing [9]. However, in the present study the milk protein yield was improved, while the potential effect of RPC on milk protein content was not supported.

Overall, RPC is fed to dairy cows during late pregnancy and early lactation in an attempt to improve lipid metabolism and health of dairy cows. In this regard, it has been assumed that RPC in the periparturient period increases the synthesis of phosphatidylcholine and VLDL, thereby improving lipid metabolism [36]. Phosphatidylcholine is a major factor affecting the rate of VLDL export and susceptibility of the liver to accumulate TAG [3]. Thus, low bioavailability of dietary choline together with the elevated requirement for VLDL export due to increased NEFA uptake poses a risk to the dairy cow developing fatty liver. Therefore, supplementing RPC to transition cows has been hypothesized to facilitate TAG secretion from the liver. Although some studies observed an effect of RPC to reduce liver TAG [16,20,25], this was not observed in all studies [6,10,11,13]. However, it has to be considered, that the average concentration of TAG varies widely in the postpartum period, including cows with extremely high concentrations, which might obscure a potential RPC-effect. Therefore, the high variability in postpartal TAG among individual cows requires the sampling of a high number of cows per treatment to be able to detect treatment effects [45].

In accordance with the results of liver TAG, the response of blood metabolites in cows to RPC supplementation has been inconsistent. While some studies found reduced blood NEFA and increased glucose concentrations [15,16,21,26,27], no overall effect of RPC on the blood metabolic profile was observed in the current meta-analysis. Possible explanations might be large variations in physiological and dietary factors, causing a high variability among treatment means. Furthermore, differences in experimental designs between the studies might be reasons for the inconsistent results. Regarding the effects of RPC on postpartal health, reduced ketosis incidence in cows fed RPC before and after calving was reported in some studies [18,36]. Elevated concentrations of BHBA and ketones might impair the functions of immune cells with possible implications on the susceptibility of dairy cows to infections [46]. Indeed, Lima et al. [36] found a reduced incidence of mastitis in dairy cows with a lower risk of ketosis due to feeding or RPC, thereby suggesting a potential benefit of improved lipid metabolism on the susceptibility of dairy cows toward mammary infections. Furthermore, reduced mastitis incidence [22] and somatic cell counts [34] in dairy cows fed RPC were observed in experiments in which no cases of ketosis were observed. However, besides the aforementioned positive effects of RPC on the incidence of diseases, several other studies found either no or mixed effects [6,11,14,37]. Lima et al. [36] suggested that RPC supplementation might be more critical during NEB and extensive lipid mobilization, as the benefits of feeding RPC on the incidence of diseases were only observed when it was fed pre- and post-calving. However, there is clearly more research needed to elucidate the possible health-promoting effects of RPC and to reveal the mechanism(s) of action of choline more precisely through a higher standardization of experiments, such as higher animal numbers, and further investigations of metabolic pathways related to lipid mobilization, liver health, and synthesis of milk constituents, among others.

In general, lipid metabolism plays a key role in the adaptation to lactation and therefore is critical to reproduction. More specifically, delayed cyclicity and reduced pregnancy rate have been reported in cows with increased blood ketone levels after parturition [36]. Therefore, feeding RPC has been hypothesized to beneficially affect reproduction, either due to a decreased risk of diseases which commonly aggravate the NEB and thus impair the BCS of dairy cows [45], or through a direct effect of choline on the reproductive tissues [36]. However, while Ardalan et al. [22] and Furken and Hoedemarker [37] found improved reproductive traits in dairy cows fed RPC, others found no or even negative effects [34,36].

Overall, no dose-dependent effects of RPC have been observed. This is supported by Baldi and Pinotti [47], showing independent effects of the dose of RPC, ranging from 6 to 60 g choline chloride per d on milk production. One reason for the missing effect of dosage could be the large variability of RPC sources used in terms of their rumen stability, as summarized in Table 2. However, separate regression analysis of the 13 studies using the same product (i.e., “Reashure”) showed no dose-dependent effects of the RPC. However, large variations in other factors such as diet formulation, mode of administration, lactation stage, and production potential might be reasons for the missing dose-dependent effects of RPC [9,47]. Response to RPC might also vary according to the supply of methyl donors such as methionine or other cofactors associated with methylation such as folates and vitamin B12 [45,47]. Therefore, further studies are required to enable statistical analysis taking into account variation among different research studies and to quantify the effects of the inclusion of dietary RPC on health and reproduction.

## 5. Conclusions

The supplementation of RPC showed positive effects on DMI and milk yield in lactating cows, while improved health status and reproduction were not consistent findings. As numerous physiological and dietary factors might be related to the responses to RPC, further research is warranted to evaluate the precise mechanisms of RPC action in lactating cows and to reveal possible health-promoting effects.

## Figures and Tables

**Table 1:** List of studies used to evaluate the effect of dietary rumen-protected choline (RPC) supplementation on dry matter intake (DMI), milk yield (MY), milk fat content (MFC), milk protein content (MPC), non-esterified fatty acids (NEFA), beta-hydroxybutyrate (BHBA), glucose and cholesterol in lactating dairy cows.

Reference	Cows/Treatment	Dose, g/d of Choline Chloride	Duration	Variables
Hartwell et al. [10]	16 16 16	0 6 12	28 d a.p. to 120 DIM	DMI, MY, MFC, MPC
Piepenbrink and Overton [11]	12 12 12 12	0 11 15 19	21 d a.p. to 63 DIM	DMI, MY, MFC, MPC, NEFA, BHBA
Pinotti et al. [12]	13 13	0 20	14 d a.p. to 30 DIM	DMI, MY, MFC, MPC, NEFA, BHBA, Glucose, Cholesterol
Zahra et al. [13]	45 45	0 14	21 d a.p. to 28 DIM	DMI, MY, MFC, MPC, NEFA, BHBA, Glucose, Cholesterol
Janovick Guretzky et al. [14]	21 21	0 15	21 d a.p. to 21 DIM	DMI, MY, MFC, MPC, NEFA, BHBA, Glucose, Cholesterol
Xu et al. [15] ^1^	7 7	0 7.5	7 d a.p. to 21 DIM.	DMI, MY, MFC, MPC, NEFA, Glucose, Cholesterol
Xu et al. [15] ^1^	9 9 9 9	0 11.3 22.5 33.8	7 d a.p. to 15 DIM	DMI, MY, MFC, MPC, NEFA, Glucose, Cholesterol
Cooke et al. [16]	12 12	0 15	45–60 d a.p. to 28–43 d a.p.	NEFA, BHBA, Glucose
Lima et al. [17]	179 179	0 15	25 d a.p. to 80 DIM	DMI, MY
Davidson et al. [18]	20 20	0 8	21 DIM to 91 DIM	DMI, MY, MFC, MPC, NEFA, BHBA, Cholesterol
Elek et al. [19,20]	16 16	0 25 a.p./50 p.p.	28 d a.p. to 60 DIM	DMI, MY, MFC, MPC, NEFA, BHBA, Glucose, Cholesterol
Chung et al. [21]	6 ^2^ 6 6	0 12.5 25	Start: 41 DIM, 10 d/period	DMI, MY, NEFA, BHBA, Glucose
Ardalan et al. [22]	10 10	0 14.4	28 d a.p. to 98 DIM	DMI, MY, MFC, MPC
Mohsen et al. [23]	12 ^3^ 12	0 15 30	Start: 56 DIM, 8d/period	DMI, MY, MFC, MPC, Glucose, Cholesterol
Suksombat et al. [24]	8 8 8	0 20 40	32 DIM to 102 DIM	DMI, MY, MFC, MPC, NEFA, BHBA, Cholesterol
Zom et al. [25]	19 19	0 14.4	21 d a.p. to 42 DIM	DMI, MY, MFC, MPC, NEFA, BHBA, Glucose
Garg et al. [26]	8 8	0 10	14–21 DIM to 104–111 DIM	DMI, MY, MFC, MPC, NEFA, Glucose, Cholesterol
Soltan et al. [27]	15 15	0 7.5	0 to 84 DIM	DMI, MY, MFC, MPC, NEFA, Glucose, Cholesterol
Rahmani et al. [28]	8 8	0 22.5	35 DIM to 63 DIM	DMI, MY, MFC, MPC
Leiva et al. [29]	11 12	0 9.4. a.p./18.8 p.p.	21 d a.p. to 45 DIM	MY, MFC, MPC, NEFA, BHBA, Glucose
Pawar et al. [30]	5 5 5 5 5	0 13.7 20.6 27.5 34.3	28 d experimental period	MY, MFC, MPC
Pineda and Cardoso [31]	25 25	0 23	Start: >80 DIM, 63 d experimental period	DMI, MY, MFC, MPC, NEFA
De Veth et al. [32]	5 ^2^ 5 5	0 12.5 25	Start: 206 DIM, 5 d/period	DMI, MY, MFC, MPC
Zhou et al. [33]	20 20	0 15	21 d a.p. to 30 DIM	DMI, MY, MFC, MPC, NEFA, BHBA, Glucose
Pirestani and Aghakhani [34]	30 30	0 15	7 d a.p. to 28 DIM	MY, MFC, MPC, BHBA
Cetin et al. [35]	8 8	0 18	21 d a.p. to 70 DIM	DMI, MY, MFC, MPC
Zenobi et al. [6]	47 46	0 17.3	17 d a.p. to 21 DIM	DMI, MY, MFC, MPC, NEFA, BHBA, Glucose

Abbreviations: a.p., ante partum; DIM, days in milk; p.p., post partum. ^1^ Two independent experiments. ^2^ Latin square design. ^3^ Switch-back design.

**Table 2:** Commercial rumen-protected choline products used in the different studies.

Product	Manufacturer	Study	Choline Chloride, % (wt/wt)	Rumen Stability, %
CapShure choline	Balchem Corp., Slate Hill, NY, USA	Hartwell et al. [10]	25	85 ^1^
Reashure	Blachem Corp., New Hampton, NY, USA	Piepenbrink and Overton [11] Zahra et al. [13] Janovic Guretzky et al. [14] Cooke et al. [16] Lima et al., [17,36] Suksombat et al. [24] Zom et al. [25] Soltan et al. [27] Furken and Hoedemaker [37] Rahmani et al. [28] De Veth et al. [32] Zhou et al. [33] Zenobi et al. [6]	25	85 ^1^ ≥60 ^2^
-	-	Xu et al. [15]	37.5	70
Overcholine 45% Coated	Ascor Chimici, Forli, Italy	Pinotti et al. [12]	45	-
Norcol-25	Nordos Italy, Bussolengo, Italy	Elek et al. [19,20]	25	20.4 ^3^
Pro-Choilne 50	Probiotech Inc., Saint-Hyacinthe, QC, Canada	Chung et al. [21]	50	50 ^4^
COL24	Soda Feed Ingredients, Monaco, France	Ardalan et al. [22]	24	-
CholiPearl	Kemin Agrifoods, South America	Leiva et al. [29] Cetin et al. [35]	18.8	-
-	Robt Morgan Inc., Paris, IL, USA	Davidsson et al. [18] Pineda and Cardoso [31]	23	28.7
-	Qingdao Worldwide International Trade Co. Ltd., Shandong, China	Mohsen et al. [23]	-	-
-	-	Garg et al. [26]	-	71.3
	Kemin Industries South Asia Pvt. Ltd., Tamilnadu, India	Pawar et al. [30]	25.4	69.2 ^5^
Soda Food Supplement	Sana Dam Pars Company, Shamsabad, Iran	Pirestani and Aghakhani [34]	25	-

^1^ In vitro analysis [38]; ^2^ After up to 72 h in situ incubation in rumen-cannulated steers [39]; ^3^ After 8 h in situ incubation in rumen-cannulated adult ewes [40]; ^4^ After 48 h in situ incubation in rumen-cannulated cows [41]; ^5^ After 24 h in situ incubation in rumen-cannulated bulls [30].

**Table 3:** Statistics for dependent variables.

Item	Studies, *n*	Values, *n*	Mean	SE	Min.	Max.	Median
DMI, kg/d	23	64	19.5	0.49	12.2	28.5	20.0
Milk yield, kg/d	26	71	32.0	1.14	10.0	48.9	32.5
Fat content, %	24	66	3.94	0.09	2.64	6.50	3.89
Fat yield, kg/d	24	66	1.20	0.05	0.36	1.82	1.27
Protein content, %	24	66	3.14	0.04	2.44	4.53	3.11
Protein yield, kg/d	24	66	0.96	0.03	0.36	1.38	1.01
NEFA, mmol/L	19	46	0.57	0.03	0.17	1.19	0.56
BHBA, mmol/L	14	36	0.78	0.08	0.15	1.86	0.57
Glucose, mg/dL	15	36	60.0	1.42	44.6	80.6	58.4
Cholesterol, mg/dL	11	28	143	6.80	78.3	210	148

Abbreviations: DMI, dry matter intake; NEFA, non-esterified fatty acids; BHBA, beta-hydroxybutyrate; SE, standard error.

**Table 4:** Dry matter intake (DMI), milk yield and constituents and blood metabolites of either control cows (CON) or cows supplemented with rumen-protected choline (RPC).

Item	CON	RPC	*n*	SEM	*p*-Value
DMI, kg/d	19.06	19.85	64	0.829	<0.001
Milk yield, kg/d	31.80	32.85	71	1.872	0.032
Fat content, %	3.843	3.873	66	0.149	0.544
Fat yield, kg/d	1.167	1.215	66	0.0754	0.012
Protein content, %	3.162	3.166	66	0.0735	0.784
Protein yield, kg/d	0.964	0.995	66	0.0569	0.054
NEFA, mmol/L	0.571	0.563	46	0.0499	0.692
BHBA, mmol/L	0.760	0.769	36	0.1149	0.744
Glucose, mg/dL	59.95	60.50	36	2.386	0.461
Cholesterol, mg/dL	145.5	146.3	28	11.47	0.862

Abbreviations: NEFA, non-esterified fatty acids; BHBA, beta-hydroxybutyrate.

**Table 5:** Dry matter intake (DMI), milk yield and constituents and blood metabolites as affected by supplementation dose of choline chloride deriving from rumen-protected choline (RPC) in lactating dairy cows.

Response Variable ^1^ (*Y*)	Parameter Estimates				Model Statistics			
	n_Treat_^2^	Intercept	SE_Intercept_	Slope	SE_Slope_	RMSE	*R* ^2^	*p*-Value
DMI, kg/d	64	19.39	0.6613	0.0141	0.0447	3.9385	0.0016	0.754
Milk yield, kg/d	71	32.94	1.5641	−0.0930	0.1016	9.6481	0.0120	0.363
Fat content, %	66	3.864	0.1187	0.0070	0.0077	0.7092	0.0129	0.364
Protein content, %	66	3.104	0.0541	0.0030	0.0035	0.3232	0.0111	0.401
NEFA, mmol/L	46	0.5490	0.0394	0.0017	0.0026	0.2023	0.0094	0.521
BHBA, mmol/L	36	0.7751	0.1004	0.0006	0.0066	0.4570	0.0002	0.932
Glucose, mg/dL	36	60.09	1.9054	−0.0116	0.1240	8.6668	0.0003	0.926
Cholesterol, mg/dL	28	143.93	8.8892	−0.1251	0.5182	36.6159	0.0022	0.811

^1^ Abbreviations: NEFA, non-esterified fatty acids, BHBA, beta-hydroxybutyrate; ^2^ n_Treat_ = number of treatment means included.

**Table 6:** Effect of rumen-protected choline (RPC) supplementation of dairy cows on postpartum disorders.

Reference	Cows/Treatment	Dose, g/d of Choline Chloride	Duration	Observed Effects of Feeding RPC
Piepenbrink and Overton [11]	12 12 12 12	0 11 15 19	21 d a.p. to 63 DIM	overall no retained placenta, numerical higher DA and ketosis in cows fed 15 g RPC; too low cow number for statistical analysis
Janovick Guretzky et al. [14]	21 21	0 15	21 d a.p. to 21 DIM	no effect on the incidence of milk fewer, metritis, DA, ketonuria, mastitis, foot/leg problems, more twinings, tendency toward more retained placenta
Lima et al. [36] ^1^	183 179	0 15	25 d a.p. to 80 DIM	lower incidence of clinical ketosis, mastitis, mastitis cases per cow, morbidity, no effect on retained fetal membranes, fever, metritis, DA, mortality
Lima et al. [36] ^1^	282 ^2^ 291 ^2^	0 15	21 d a.p. to parturition	no effect on clinical ketosis, mastitis, lower number of mastitis cases per cow, no effect on DA, trend toward higher morbidity due to increased incidence of metritis and fever, higher incidence of retained fetal membranes
Davidson et al. [18]	20 20	0 40	21 DIM to 91 DIM	lower ketosis incidence in multiparous cows
Ardalan et al. [22]	10 10	0 14.4	28 d a.p. to 98 DIM	Numerical lower incidence of mastitis, retained placenta, uterine problems, and dystocia; overall no ketosis, DA, milk fever and foot/leg problems
Furken and Hoedemaker [37]	149 149	0 14	21 d a.p. to 21 DIM	Lower incidence of subclinical endometritis, higher sickness rates after day 100 postpartum
Zhou et al. [33]	20 20	0 15	21 d a.p. to 30 DIM	Numerical lower incidence of ketosis; no effect on displaced abomasum, retained placenta and mastitis
Pirestani and Aghakhani [34]	30 30	0 15	7 d a.p. to 28 DIM	lower somatic cell count; overall no ketosis, mastitis, lameness, dystocia
Zenobi et al. [6]	47 46	0 17.3	17 d a.p. to 21 DIM	no effect on fever, uterine diseases, hyperketonemia, ketosis, mastitis, digestive upsets, morbidity

Abbreviations: a.p., ante partum; DA, displaced abomasum; DIM, days in milk; p.p., postpartum. ^1^ Two independent experiments. ^2^ Only primiparous cows.

**Table 7:** Effect of rumen-protected choline (RPC) supplementation of dairy cows on reproductive performance.

Reference	Cows/Treatment	Dose, g/d of Choline Chloride	Duration	Observed Effects of RPC Supplementation
Ardalan et al. [22]	10 10	0 14.4	49 d a.p. to 98 DIM	no effect on days to first estrus, open days and services per conception, numerical higher number of pregnant cows (60% vs. 30%)
Furken and Hoedemaker, [37]	149 149	0 14	21 d a.p. to 21 DIM	higher number of cyclic cows in week 5 postpartum (50% vs. 41%)
Lima et al. [36] ^1^	183 179	0 15	25 d a.p. to 80 DIM	no effect on reproduction (conception rate, percentage of cows cycling, pregnancy loss) but interactive effects between RPC and parity: primiparous cows receiving RPC: less likely to be cycling at 61 DIM (64% vs. 71%), multiparous cows receiving RPC: opposite effect (93% RPC-cows vs. 86% CON-cows cycling)
	282 ^2^ 291 ^2^	0 15	21 d a.p. to parturition	no effect on reproduction (conception rate, pregnancy loss)
Pirestani and Aghakhani [34]	30 30	0 15	7 d a.p. to 28 DIM	lower service/conception rate (1.8 vs. 2.5), longer time of calving to first service (67 d vs. 61 d) and calving to first visible oestrous (62 d vs. 54 d)

Abbreviations: a.p., ante partum; DIM, days in milk; p.p., postpartum. ^1^ Two independent experiments. ^2^ Only primiparous cows.
